# Impact of COVID-19 pandemic on medical postgraduate training in the United States

**DOI:** 10.1080/10872981.2020.1774318

**Published:** 2020-06-04

**Authors:** Ehizogie Edigin, Precious Obehi Eseaton, Hafeez Shaka, Pius Ehiremen Ojemolon, Iriagbonse Rotimi Asemota, Emmanuel Akuna

**Affiliations:** a Department of Internal Medicine, John H Stroger Jr. Hospital of Cook County, Chicago, IL, USA; b Department of Internal Medicine, University of Benin Teaching Hospital, Benin, Nigeria; c Department of Anatomical Sciences, St. George’s University, St. George’s, West Indies

**Keywords:** Postgraduate medical education, COVID-19, pandemic, medical residents, medical fellows

The COVID-19 pandemic has affected almost all areas of human endeavors. The impact of the pandemic on medical students’ education in the USA (US) has been well established in the literature [1–3]. However, the impact of COVID-19 on medical postgraduate training of residents and fellows in the US has not been adequately discussed in the literature. On 17 March 2020, the Association of American Medical Colleges (AAMC) issued guidance to temporarily suspend medical student clinical clerkships due to the COVID-19 pandemic [4]. However, residents and fellows in postgraduate training programs in the US have continued to carry out their clinical duties. The COVID-19 pandemic has however changed the postgraduate training experience in many ways.

Outpatient volume has drastically reduced during this pandemic. Non-urgent outpatient clinic appointments have been canceled. These visits are now being conducted over the phone or through video calls. These telemedicine visits do not include a traditional physical exam. Physical examination is an essential skill and can only be mastered by practice. The non-COVID patient volume on the inpatient services of some hospitals has also markedly reduced. This markedly reduced patient volume in the inpatient service has limited trainee education. Medical trainee education is consolidated by a high volume of patient encounters. A high patient volume allows trainees to see atypical presentations of common diseases and rare diseases.

The reduced patient volume has reduced opportunities for trainees to perform essential inpatient procedures. Some residency programs had to waive the minimum number of procedures required for 3^rd^-year internal medicine residents to graduate because of the COVID-19 pandemic. These residents may graduate and become attendings without achieving proficiency in essential medical procedures. Most hospitals have canceled outpatient/elective surgeries during the COVID-19 pandemic. Trainees in procedural specialties are affected by this. This imparts on the trainee’s ability to develop proficiency in these outpatient procedures.

In addition to trainees working with a reduced patient volume, there has been a reduction in the diversity of disease pathologies exposed to trainees during this COVID-19 crisis. Many hospitals have created special COVID services. Trainees are spending a considerable amount of time in these COVID services which expose trainees to only patients primarily managed for COVID-19. During the pre-COVID era, an internal medicine resident on a typical day will likely see a broad spectrum of cases ranging from simple cellulitis to life-threatening acute coronary syndrome. The focus of the trainee has shifted to COVID-19. Historically postgraduate medical training was well rounded. If this paradigm shift continues for too long, trainees in medical specialties may lose or never develop skills in managing a broad range of medical pathologies.

Due to the COVID-19 pandemic and need to limit exposure of healthcare workers, some trainees have been placed on standby. This means that trainees are at home and only come to the hospital if the need arises. The period of postgraduate training is a short time to master the skills that are needed for independent practice. More trainees in specialized non-primary care specialties are on standby due to markedly reduced patient volume in these specialties. Although having a group of trainees on stand-by will reduce healthcare worker exposure to COVID, this will reduce the learning opportunities for trainees to become competent in their respective fields.

All major in-person academic conferences have been canceled during this COVID-19 era. Academic conferences allow trainees to present their research findings in the form of oral or poster presentations. These opportunities can advance the careers of residents who wish to pursue further training in competitive subspecialties. Most of these conferences have decided to switch from traditional in-person gatherings to a virtual platform to provide educational content safely. While trainees may still be able to present their research findings through this virtual path, trainees lose the opportunity to network with experts in their desired fields. These networking opportunities are much easier to harness during face-to-face events.

Seven thousand three hundred and seventy-six international medical graduates (IMG) obtained first-year residency positions in the 2020 match and are expected to begin postgraduate residency training on 1 July 2020. Four thousand two hundred and twenty-two of these IMGs are non-US citizens [5]. Many of these non-US citizen IMGs are currently outside of the US and will need visa sponsorship to enter the US and begin residency training. The COVID-19 pandemic has led to travel and visa restrictions in the US and abroad. If these travel and visa restrictions cause a delay in new trainees from commencing work on time, there may be a potential shortage of medical personnel available to care for patients on 1 July 2020. This shortage will place more workload and potentially worsen the educational experience for the remaining doctors in training.

The COVID-19 pandemic has the potential to affect the recruitment process for postgraduate training positions. Every year thousands of medical students and doctors travel from all over the world to interview for first-year residency positions across different teaching hospitals in the US. Thousands of resident physicians in the US also do the same to obtain postgraduate fellowship positions. Due to the potential healthcare risk associated with travel, the AAMC strongly encourages teaching hospitals to conduct all interviews with potential residents and fellows in a virtual setting, either through phone calls or video conferencing [6]. The effectiveness of recruiting trainees in this virtual setting has not been established. However, in the current climate, teaching hospitals may choose this route as the safest option available.

In conclusion, residents and fellows are both hospital employees and postgraduate students in training. While patient care and clinical responsibilities are important, the educational experience of the trainees should not be de-emphasized. Didactics via virtual platforms should continue. Trainees on standby should be encouraged to do research and scholarly activities. Faculty should continue to work on curriculum development using telemedicine and other creative avenues to ensure the delivery of the latest educational materials to trainees.
